# A molecular systems approach to modelling human skin pigmentation: identifying underlying pathways and critical components

**DOI:** 10.1186/s13104-015-1128-6

**Published:** 2015-04-29

**Authors:** Arathi Raghunath, Awanti Sambarey, Neha Sharma, Usha Mahadevan, Nagasuma Chandra

**Affiliations:** Molecular Connections Private Limited, Bangalore, 560004 India; Department of Biochemistry, Indian Institute of Science, Bangalore, 560012 India

**Keywords:** Systems perspective, Skin, UV, Melanin, Networks

## Abstract

**Background:**

Ultraviolet radiations (UV) serve as an environmental stress for human skin, and result in melanogenesis, with the pigment melanin having protective effects against UV induced damage. This involves a dynamic and complex regulation of various biological processes that results in the expression of melanin in the outer most layers of the epidermis, where it can exert its protective effect. A comprehensive understanding of the underlying cross talk among different signalling molecules and cell types is only possible through a systems perspective. Increasing incidences of both melanoma and non-melanoma skin cancers necessitate the need to better comprehend UV mediated effects on skin pigmentation at a systems level, so as to ultimately evolve knowledge-based strategies for efficient protection and prevention of skin diseases.

**Methods:**

A network model for UV-mediated skin pigmentation in the epidermis was constructed and subjected to shortest path analysis. Virtual knock-outs were carried out to identify essential signalling components.

**Results:**

We describe a network model for UV-mediated skin pigmentation in the epidermis. The model consists of 265 components (nodes) and 429 directed interactions among them, capturing the manner in which one component influences the other and channels information. Through shortest path analysis, we identify novel signalling pathways relevant to pigmentation. Virtual knock-outs or perturbations of specific nodes in the network have led to the identification of alternate modes of signalling as well as enabled determining essential nodes in the process.

**Conclusions:**

The model presented provides a comprehensive picture of UV mediated signalling manifesting in human skin pigmentation. A systems perspective helps provide a holistic purview of interconnections and complexity in the processes leading to pigmentation. The model described here is extensive yet amenable to expansion as new data is gathered. Through this study, we provide a list of important proteins essential for pigmentation which can be further explored to better understand normal pigmentation as well as its pathologies including vitiligo and melanoma, and enable therapeutic intervention.

**Electronic supplementary material:**

The online version of this article (doi:10.1186/s13104-015-1128-6) contains supplementary material, which is available to authorized users.

## Background

Skin pigmentation is a phenotype evolved and retained over generations, primarily as protection against harmful Ultraviolet (UV) radiations [1]. Melanin, a light-absorbing polymer with photochemical properties imparts colour to human skin and other tissues such as hair and iris, and provides protection against UV induced damage. Variations in skin pigmentation depend on the level and the type of melanin expressed. The two major forms of melanin expressed in humans are eumelanin and pheomelanin, imparting black or brown and red haired or freckled phenotypes respectively. Melanin is synthesized in melanocytes in specialized organelles called melanosomes [2]. Transfer of the pigment to keratinocytes that constitute the upper layers of the epidermis is essential for phenotypic manifestation of skin pigmentation. The dendrites on melanocytes aid in the transfer of melanosomes to keratinocytes where melanosome uptake occurs through phagocytosis [2].

Exposure to UV radiation triggers signalling cascades in keratinocytes, which in turn induce autocrine signalling as well as paracrine signalling with neighbouring melanocytes resulting in melanogenesis, and thereby subsequent processes leading to skin pigmentation. While UV radiations trigger pigmentation, they are also capable of causing skin cancer due to the generation of free radicals and DNA damage, which is actively prevented by the ‘epidermal melanin unit’ - the association of one melanocyte with several surrounding epidermal keratinocytes [3]. UV exposure can also lead to the depletion of folic acid, though a certain amount of exposure is essential for Vitamin D synthesis [4]. Hence, a balanced exposure to UV radiation is critical, and is maintained by the regulation of melanin synthesis. Constitutive skin pigmentation is determined by the genetic constitution of the individuals while facultative pigmentation is governed by environmental factors [5]. Oxidation and re-distribution of melanin in skin induces some marginal protection but delayed and sustained tanning response to UV includes melanin synthesis and transport to keratinocytes [6].

The process of UV mediated skin pigmentation is complex and dynamic, involving various cell types and extensive cross-talk between them as well as several intracellular signalling cascades. While several studies over the past few decades have provided insights into melanogenesis, melanosome formation and pigmentation [7-9] their focus has been primarily on individual reactions and molecular changes. The World Health Organization reports incidences of between 2 and 3 million non-melanoma skin cancers and more than 100,000 melanoma skin cancers globally each year [10]. These statistics highlight the need for further studies in this area, and a wholistic understanding of pigmentation is thus necessary.

To this end, a network approach is useful as networks have emerged as an important tool to study biological phenomena [11]. The ‘omics’ era has resulted in the generation of enormous biological data, from individual gene and protein sequences and structures to well-defined physical and functional interactions and elaborate signalling cascades. By integrating this information of communication between various molecular components in the form of an interaction network, biological processes can be represented and analysed at a systems level, wherein the contributions and effects of individual components and interactions on the entire system can be studied to generate insights which cannot be achieved by reductionist approaches alone. Extensive research in the field of skin pigmentation has resulted in vast accumulation of data, but studies that integrate and analyse this data are limited [12-14].

In this study, we curate data available from literature and construct a comprehensive network model of UV mediated pigmentation in both keratinocytes and melanocytes, in an attempt to provide mechanistic insights into signalling cascades underlying skin pigmentation. Further, we study the effects of knock-outs of certain components on these signalling pathways, leading to the identification of essential genes as well as highlight key destinations and possible alternative signalling routes in the process of pigmentation.

## Results

### A comprehensive network model of human pigmentation

We have constructed a network model of human skin pigmentation based on extensive data available from literature. The model includes various components of (a) melanin biosynthesis, (b) melanosome biogenesis, (c) dendrite formation and (d) uptake of melanosomes by keratinocytes. Such a comprehensive systems-level picture of skin pigmentation has the potential to address several questions, particularly the critical nature of various factors involved in pigmentation and its response to UV radiation.

Several experimental studies have been referred to for network construction as enlisted in Additional file 1. However, for clarity, a brief description of the model and its components is given below.

Melanin, the photosensitive polymer that imparts skin pigmentation is synthesised and stored in melanosomes - organelles specific to melanocytes [7]. Melanin is synthesised in the body from the amino acid tyrosine, through a series of biochemical reactions primarily involving the enzymes tyrosinase, DOPAchrome tautomerase and tyrosinase-related protein 1 (TYRP1). Tyrosine is first oxidized to DOPA and subsequently to DOPAquinone by the enzyme tyrosinase. DOPAquinone can be enzymatically converted to either eumelanin or pheomelanin. During eumelanin synthesis, DOPAquinone is converted to leucoDOPA and subsequently to DOPAchrome by auto-oxidation. DOPAchrome tautomerase, in the presence of dihydroxyindole-2-carboxylic acid oxidase, converts DOPAchrome to 5,6-dihydroxy indole. Tyrosinase acts on 5,6-hydroxy indole finally resulting in eumelanin synthesis. An alternate biosynthesis pathway leading to pheomelanin involves conversion of DOPAquinone to cysteinylDOPA in presence of glutathione/cysteine, followed by oxidative polymerization of the benzothiazinylalanine intermediates, resulting in pheomelanin synthesis [15].

Though melanin biosynthesis is a central process in skin pigmentation, multiple associated pathways are necessary for successful manifestation of the phenotype. In response to the necessary UV stimulus, melanocytes form dendrites, and through them transport melanin carrying melanosomes to the peripheral keratinocytes where the melanosomes are subjected to phagocytosis, resulting in the expression of melanin in keratinocytes, ultimately leading to skin pigmentation [7].

**Figure 1:**
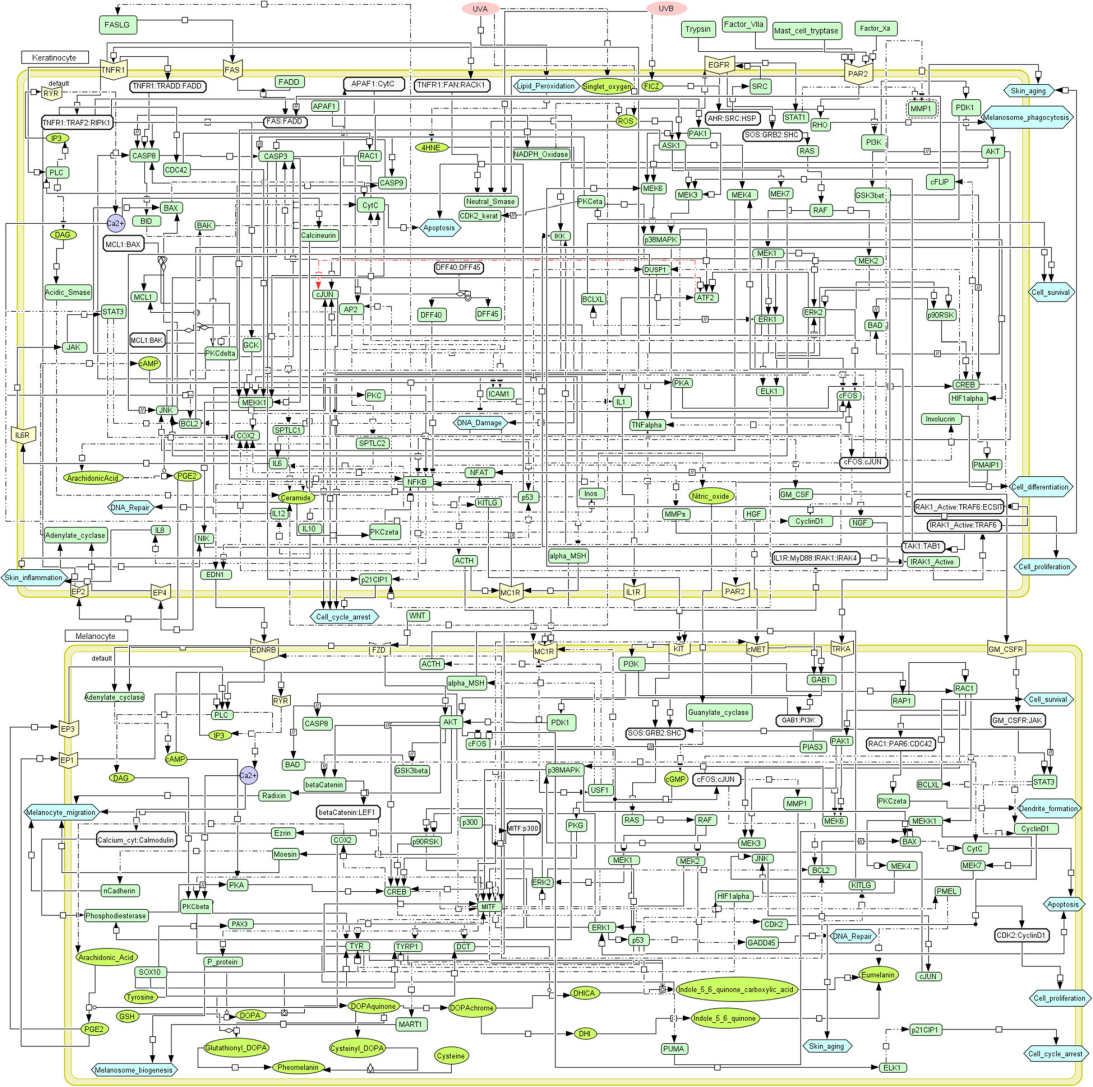
Schematic representation of signalling events triggered in skin cells upon exposure to UV radiation. The two compartments in the image represent the two cell types- keratinocyte and melanocyte. Generic proteins are represented in rectangular boxes while receptors are specifically shown on the membrane; biological processes are represented as hexagons, and secondary messengers and other small molecules involved in the signaling network as ellipses and circles. The environmental triggers UVA and UVB are represented as pink ellipses. Complexes of two or more proteins are also depicted in rectangles, with the proteins involved in the complex separated by a colon (:). The arrows describe the nature of interaction: activation is depicted as , inhibition as , expression as  and third molecule regulation as . The schema also describes paracrine (reactions occurring within a compartment) and autocrine (reactions occurring cross-compartmentally) signalling.

Here, we present an extensive systems model of pigmentation that includes interactions and pathway networks involved in all the major biological processes discussed above leading to the pigmentation of skin, beginning with melanosome formation and melanogenesis and culminating in its expression in keratinocytes leading to skin pigmentation. Figure 1 provides a schematic representation of the complex network of the various processes leading to human skin pigmentation. The model is depicted in the form of a static map that is compartmentalised to depict several signalling events and flow of signal information in keratinocytes and melanocytes on exposure to UV. As can be observed, the model captures several signalling cascades triggered in keratinocytes on initial exposure such as lipid peroxidation, MAPK signalling, TNF-receptor mediated skin inflammation and other processes which result in melanogenesis in melanocytes via extensive cross-talk. The model also contains a detailed molecular description of the synthesis of melanin in melanosomes, followed by dendritogenesis which enables melanosome phagocytosis by keratinocytes, where melanin finally manifests itself in the form of the skin pigment. The model also includes additional pathways stimulated by UV radiation like skin ageing, cell proliferation and differentiation and DNA damaged- minimised by the process of DNA repair, mediated by melanin. While the model provides a comprehensive description of skin pigmentation, given the vastness of research in this field and increasing data availability, it lends itself to incorporation of additional data as and when it becomes available.

Based on the signalling events described in the model, 265 participating molecules were identified, including 189 proteins, 33 small molecules/compounds, 23 complexes, 18 biological processes and 2 environmental factors. The model thus incorporates multiple levels of detail. For these 265 molecules or nodes, interactions were extracted as described in Methods, and a network was constructed comprising 265 nodes and 429 edges. The edges were assigned direction by extensive literature curation, highlighting the functional nature of each interaction. 142 nodes were involved in keratinocyte signalling, while 113 nodes contributed to signalling within melanocytes, and these were ascribed the suffix ‘kerat’ and ‘melan’ respectively. The complete list of all nodes and edges in the network along with the nature and direction of their interactions are provided in Additional file 1. A sample network description has been provided in Table 1. This network was visualized in Cytoscape v3.1.0 and several network properties were computed based solely on network topology. As the network was directed, both in-degree and out-degree distributions were computed describing their connectivity and certain ‘hub’ nodes which are central to the network were thus identified. All network properties of degree distributions, identified hubs, node centrality, edge centrality and radiality computed are described in Additional file 2.

**Table 1:** **A sample Protein-Protein interaction network constructed in this study**

**Node A**	**Node B**	**Interaction type A- > B**
UVA	Lipid_Peroxidation_kerat	induces
Lipid_Peroxidation_kerat	4HNE_kerat	increases level
4HNE_kerat	DNA_Damage_kerat	induces
DNA_Damage_kerat	TP53_kerat	activates
TP53_kerat	ACTH_kerat	increases expression
TP53_kerat	alpha_MSH_kerat	increases expression
ACTH_kerat	MC1R_melan	activates
alpha_MSH_kerat	MC1R_melan	activates
MC1R_melan	ADCY4_melan	activates
ADCY4_melan	cAMP_melan	increases level
CREB1_melan	PTGS2_melan	increases expression
PGE2_kerat	PTGER3_melan	activates
PGE2_kerat	PTGER1_melan	activates
PTGER3_melan	PLC_melan	activates
PTGER1_melan	PLC_melan	activates
PLC_melan	DAG_melan	increases level
DAG_melan	PRKCB_melan	activates
PTGS2_melan	PGE2_melan	Increases level
PRKCB_melan	TYR_melan	activates
TYR_melan	Eumelanin_melan	Increases level

The complete network can be found in Additional file 1.

### Identifying biochemical routes in the network among defined sources and targets

**Figure 2:**
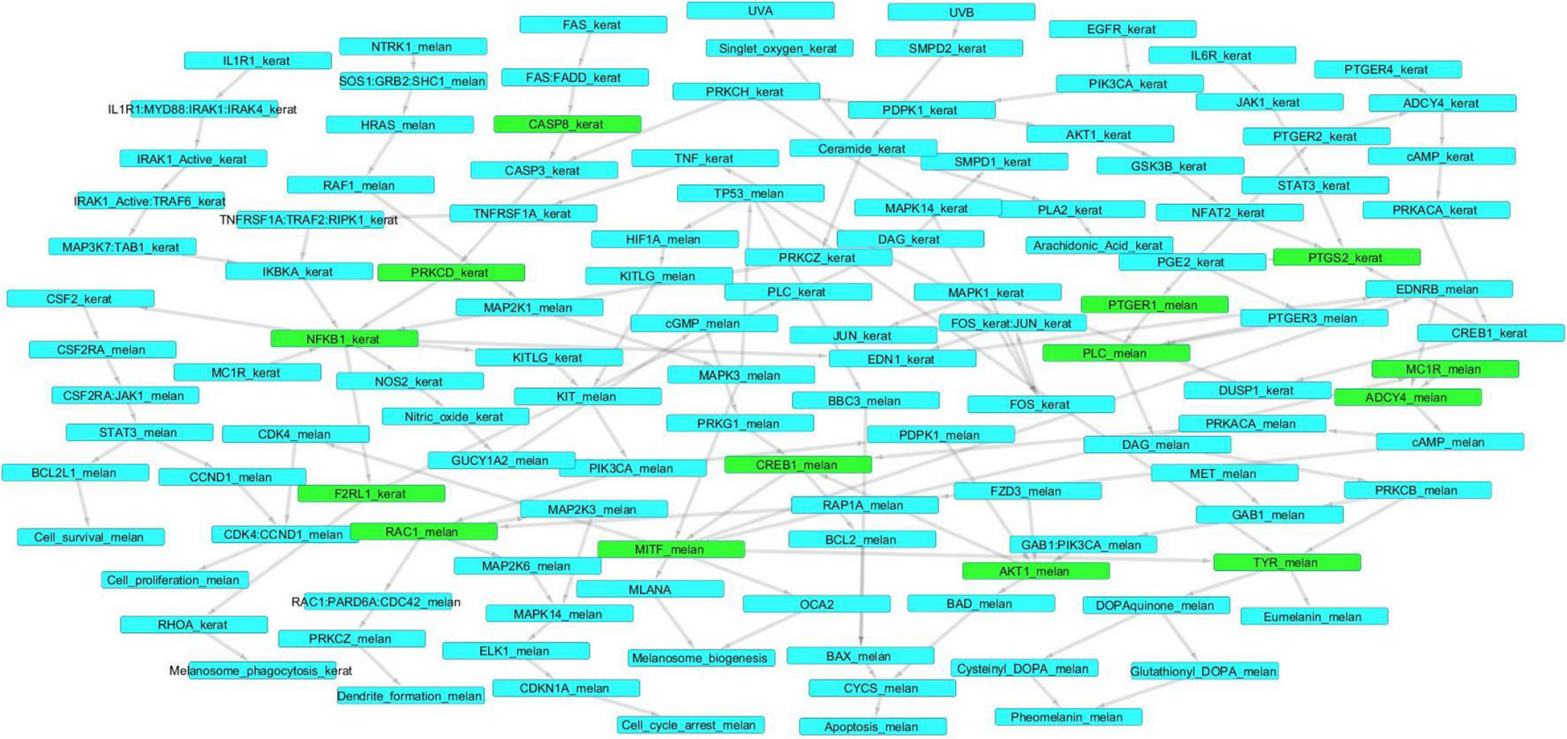
Directed network of paths from selected source to target nodes (S2T). Arrows indicate direction of interaction. Paths from source to target nodes can be traced. Nodes identified as ‘essential’ post perturbation analysis are highlighted in green.

The constructed network provides a basis for understanding communication and signalling within the system. A systems level view of pigmentation provides insights into how information flows via signalling cascades both at inter-and intra cellular levels, and encompasses cross talk among different molecules and cell types. One approach towards understanding network dynamics and communication is through the analysis of shortest paths between pairs of nodes, as demonstrated in several studies in different biological conditions [16,17]. A total of 33,779 all-vs-all paths for the 265 nodes were computed. For a more definitive analysis of pathways of interest in the process of pigmentation, specific source and destination nodes were chosen and paths from these sources to destinations were inspected in detail. The initiating nodes or sources included key receptors and triggers such as UV radiation. 20 source nodes were identified, based on their likelihood of initiating signalling events. All sources culminated in biological end processes such as melanosome formation, melanogenesis, dendrite formation and melanosome phagocytosis, among others. A total of 9 destinations were thus shortlisted. Table 2 enlists the selected source and target nodes in the network. Between these 20 sources and 9 destination nodes, 157 paths were computed which were further analyzed. The source to target (S2T) sub-network had 127 nodes and 150 edges, and the resulting network was completely interconnected, with no independent interactions. This indicates a well-knit network with extensive signalling crosstalk. The 127 nodes were seen to participate in various biological processes in both keratinocytes and melanocytes including cell differentiation, proliferation, survival, cell cycle arrest, apoptosis, melanosome biogenesis, eumelanin and pheomelanin synthesis, melanocyte migration, dendrite formation, melanosome phagocytosis, skin inflammation and skin ageing, thus covering and connecting several signalling cascades, as enlisted in Additional file 3. This S2T sub network was visualized in Cytoscape and is depicted in Figure 2. All computed paths from the selected source to target nodes are listed in Additional file 4.

**Table 2:** **Nodes selected as source and target for shortest path analysis**

**Source nodes**	**Target nodes**
MET_melan	Eumelanin_melan
EDNRB_melan	Pheomelanin_melan
EGFR_kerat	Dendrite_formation_melan
PTGER1_melan	Melanosome_phagocytosis_kerat
PTGER2_kerat	Apoptosis_melan
PTGER3_melan	Cell_proliferation_melan
PTGER4_kerat	Melanosome_biogenesis
FAS_kerat	Cell_survival_melan
FZD3_melan	Cell_cycle_arrest_melan
CSF2RA_melan	
IL1R1_kerat	
IL6R_kerat	
KIT_melan	
MC1R_kerat	
MC1R_melan	
F2RL1_kerat	
TNFRSF1A_kerat	
NTRK1_melan	
UVA	
UVB

### Identification of novel signalling pathways

Of the 157 computed paths between the desired source and target nodes, a few of them were shown to be well characterised in literature, thus serving to validate our analysis. Signalling through Protein Kinase A (PKA) culminating in melanogenesis has been well established. Also, reports of melanocyte stimulating hormone (alphaMSH/MC1R) mediated melanogenesis involving PKA/CREB signalling have been elucidated in a few individual studies [18,19]. Through our study, we identified the same pathway and traced it to MSH as the source. Such a study helps us to systematically relate individual genes to particular processes.

Another example pathway identified in our network describes the stem cell factor (KITLG) mediated activation of MITF, a primary transcription factor that regulates expression of melanogenic proteins.

Several novel signalling cascades showing possible paracrine signalling between keratinocytes and melanocytes were predicted by the network. As an example, some of the predicted UV mediated paths that lead to melanin synthesis, dendrite formation, phagocytosis and melanocyte proliferation are described in Table 3. Certain observations from literature are also provided which could possibly validate the existence of such biological routes.

**Table 3:** **Example predicted pathways from source to targets**

**Biological process**	**UV mediated melanogenesis [Tyrosinase activation]-paracrine effect**
Predicted pathway	UV- > Singlet_oxygen_kerat- > Ceramide_kerat- > PLA2_kerat- > Arachidonic_Acid_kerat- > PGE2_kerat- > PTGER3_melan- > PLC_melan- > DAG_melan- > PRKCB_melan- > TYR_melan- > Eumelanin_melan/ Pheomelanin_melan
Validation	PGE2 acts as a ligand for PTGER3 receptor. COX2 is the enzyme involved in the conversion of arachidonic acid to PGE2. UV induced COX2 and increased PGE2 production in keratinocytes [42-45].
Predicted pathway	UV- > Singlet_oxygen_kerat- > Ceramide_kerat- > PRKCZ_kerat- > NFKB1_kerat- > PTGS2_kerat
Validation	Antioxidants like Astaxanthin are shown to inhibit UV induced PGE2 production possibly by down regulating COX2 expression [46].
**Biological process**	**UV mediated melanogenesis [Tyrosinase expression]-paracrine effect**
Predicted pathway	UV- > Singlet_oxygen_kerat- > Ceramide_kerat- > PRKCZ_kerat- > NFKB1_kerat- > KITLG_kerat - > KIT_melan- > PIK3CA_melan- > PDPK1_melan- > AKT1_melan- > CREB1_melan- > MITF_melan- > TYR_melan- > Eumelanin_melan/ Pheomelanin_melan
Validation	KIT mediated regulation of MITF activity is shown to involve PI3K/AKT signaling [47].
Predicted pathway	UVA- > Singlet_oxygen_kerat- > Ceramide_kerat- > PRKCZ_kerat- > NFKB1_kerat- > NOS2_kerat- > Nitric_oxide_kerat- > GUCY1A2_melan- > cGMP_melan- > PRKG1_melan- > CREB1_melan- > MITF_melan- > TYR_melan- > melanogenesis
Validation	C-phycocyanin, a phycobiliprotein from spirulina that has antioxidant function inhibits melanogenesis by decreasing activity of CREB and suppressing tyrosinase expression [48]. Compound, MHY498 inhibited sodium nitroprusside (SNP, a NO donor)-induced NO generation, and suppressed tyrosinase expression, MITF stimulation and melanin synthesis through cGMP-mediated signaling pathway in B16F10 melanoma cells [49].
**Biological process**	**UV mediated alpha MSH production**
Predicted pathway	UVA- > Lipid_Peroxidation_kerat- > 4HNE_kerat- > DNA_Damage_kerat- > TP53_kerat- > alpha_MSH_kerat
Validation	UV releases singlet oxygen upon lipid peroxidation [50]. N-acetylcysteine with antioxidant properties can suppress alphaMSH and ACTH production in response to UV [51]. AlphaMSH is synthesised in keratinocytes in a p53 dependent manner [52].
**Biological process**	**UV mediated melanocyte dendrite formation-paracrine effect**
Predicted pathway	UV- > Singlet_oxygen_kerat- > Ceramide_kerat- > PRKCZ_kerat- > NFKB1_kerat- > KITLG_kerat- > KIT_melan- > PIK3CA_melan- > RAC1_melan- > RAC1:PARD6A:CDC42_melan- > PRKCZ_melan- > Dendrite_formation_melan
Validation	UVB exposure increased tree branch-like dendrites and activated Rac1 in a time-dependent manner in B16 melanoma cells [53]. Transiently expressed Rac1 activated mutants induces the formation of dendrite-like structures in human melanoma cell lines [34].
**Biological process**	**UV mediated NFKB1 activation and secretion of paracrine factors**
Predicted pathway	UV- > Singlet_oxygen_kerat- > Ceramide_kerat- > PRKCZ_kerat- > NFKB1_kerat- > EDN1_kerat
Predicted pathway	UV- > Singlet_oxygen_kerat- > Ceramide_kerat- > PRKCZ_kerat- > NFKB1_kerat- > CSF2_kerat
Predicted pathway	UV- > Singlet_oxygen_kerat- > Ceramide_kerat- > PRKCZ_kerat- > NFKB1_kerat- > KITLG_kerat
Validation	UV is known activate NFkappaB in keratinocytes [54,55].
**Biological process**	**UV mediated melanocyte proliferation-paracrine effect**
Predicted pathway	UVA- > Singlet_oxygen_kerat- > Ceramide_kerat- > PRKCZ_kerat- > NFKB1_kerat- > CSF2_kerat- > CSF2RA_melan- > CSF2RA:JAK1_melan- > STAT3_melan- > CCND1_melan- > CDK4:CCND1_melan- > Cell_proliferation_melan
Validation	Korean Red Ginseng extract or its saponin (KRGE or SKRG) decreased the expression of CSF2 in keratinocytes induced by UVB irradiation and also decreased proliferation of melanocytes [56].
**Biological process**	**UV induced melanosome phagocytosis**
Predicted pathway	UVA- > Singlet_oxygen_kerat- > Ceramide_kerat- > PRKCZ_kerat- > NFKB1_kerat- > F2RL1_kerat- > RHOA_kerat- > Melanosome_phagocytosis_kerat
Validation	In a co-culture system model constructed using the primary human melanocytes and keratinocytes, increased melanosome transfer and also upregulation of F2RL1 protein in the keratinocytes is observed when treated with low concentrations of H(2)O(2) [57]. Madecassoside (MA), a pentacyclic triterpene significantly inhibited UVR-induced melanin synthesis and melanosome transfer in a co culture system of keratinocytes and melanocytes and also inhibited F2RL1 expression in keratinocytes [58].

Validation refers to observations from literature that could possibly support the existence of such pathways.

### Perturbation networks: identifying ‘essential nodes’

Interaction networks help identify important nodes based on their connections and describe the topological architecture of a system. In order to assess the relative importance of a node, the following criteria was used: a) node degree, leading to the identification of hubs in the network (b) frequency of occurrence of a given node in all shortest paths – the more frequently a node occurs, the greater is its importance in the system as it is an important mediator in signalling from several sources to their respective destinations.

Based on these criteria, 33 nodes were shortlisted as most important or significant in the network. While this result is significant in itself, our objective was to identify the functional effects of these molecules in the process of pigmentation. These nodes were then knocked-out and 33 different perturbation networks were constructed, with each network specific to a perturbation wherein a selected node and its connecting edges were removed. Shortest paths from the same 20 sources to the 9 destinations were computed for each perturbed network and analyzed to identify all plausible paths leading to a similar end process.

**Figure 3:**
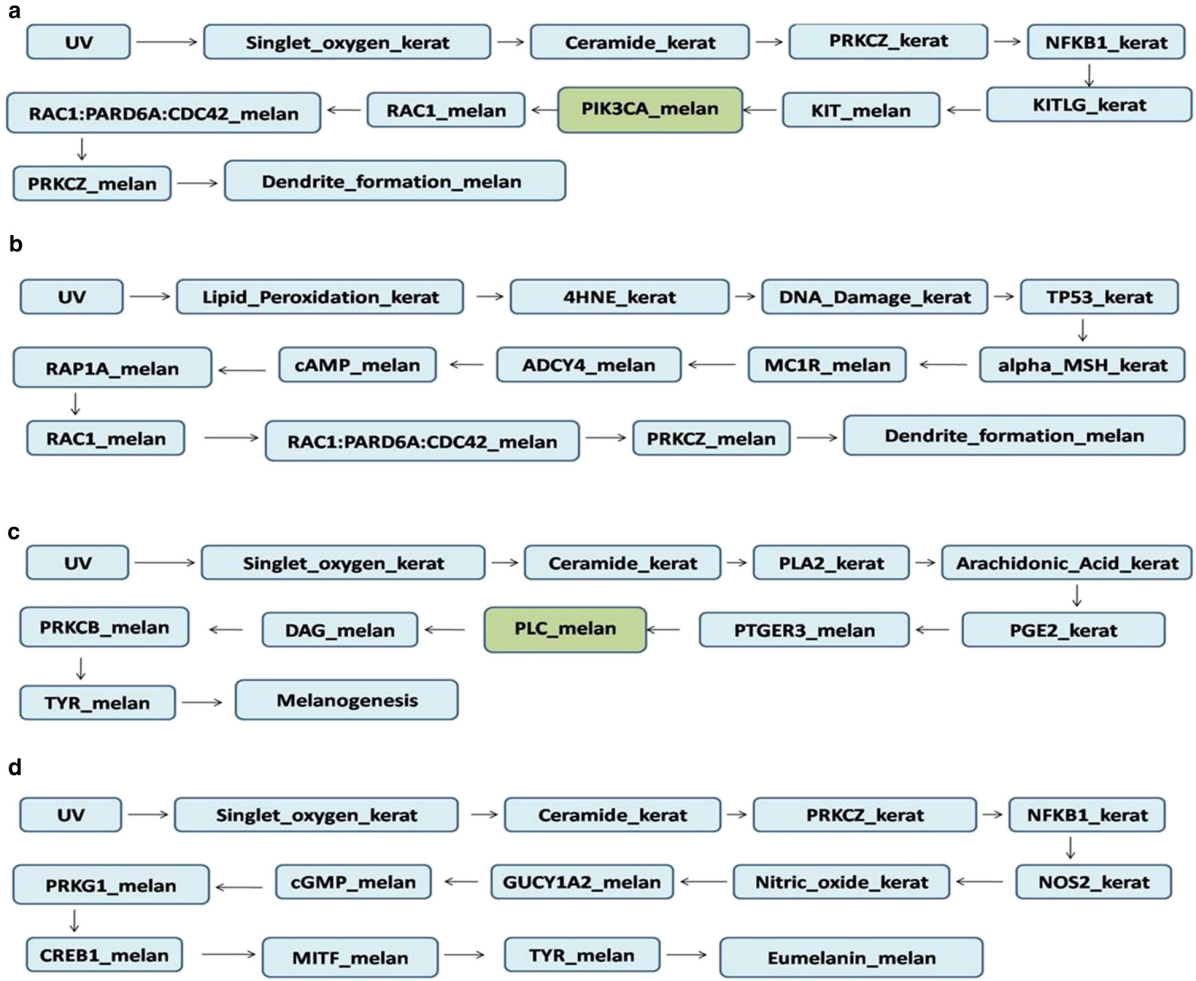
Effect of perturbation on UV mediated dendrite formation and melanogenesis: Alternate paths taken post node knockouts. The perturbed nodes are highlighted in green. **(a)** & **(b)** pathway of UV mediated dendrite formation in melanocytes before and after PIK3CA_melan knockout **(c)** & **(d)** UV mediated melanogenesis before and after PLC_melan knockout.

Perturbation studies served to address the following questions: (a) does a signal from a given source reach its destination if the node mediating its pathway is perturbed? and (b) do alternate paths exist from a particular source to a destination that can be utilised upon perturbation? While some perturbations resulted in the complete lack of signalling from a given source to a target, several others resulted in signalling via alternate routes. Nodes whose absence resulted in abrogation of pathways were considered as ‘essential nodes’. In certain cases, two or more nodes are seen to be essential for a given pathway. Table 4 shortlists these essential nodes, rank listed in order of the number of pathways they disrupt, and these nodes are also highlighted in Figure 2. A few examples of alternate routes upon perturbation are provided in Table 5, and a few others illustrated in Figure 3. Studies from literature supporting the possibility of such alternate paths have also been cited, and none of these studies overlap with the literature referred to while building the model, thereby providing greater credibility to our findings. All perturbation results describing essential nodes as well as alternate paths have been provided in Additional file 5.

**Table 4:** **Essential nodes - Nodes are ranked according to the number of source- > target pathways they are essential in**

**Node**	**No: of source- > target pathways it is essential in**
RAC1_melan	34
MITF_melan	29
NFKB1_kerat	25
TYR_melan	18
PLC_melan	14
PTGS2_kerat	9
CASP8_kerat	9
F2RL1_kerat	8
AKT1_melan	7
ADCY4_melan	7
CREB1_melan	4
PRKCD_kerat	2
PTGER1_melan	1
MC1R_melan	1

**Table 5:** **Alternate paths taken up post perturbation**

**Biological process perturbed gene**	**UV induced melanin synthesis PTGER3_melan**
Control Path	UV- > Singlet_oxygen_kerat- > Ceramide_kerat- > PLA2_kerat- > Arachidonic_Acid_kerat- > PGE2_kerat- > PTGER3_melan- > PLC_melan- > DAG_melan- > PRKCB_melan- > TYR_melan- > Melanogensis
Alternate Path	UV- > Singlet_oxygen_kerat- > Ceramide_kerat- > PRKCZ_kerat- > NFKB1_kerat- > EDN1_kerat- > EDNRB_melan- > PLC_melan- > DAG_melan- > PRKCB_melan- > TYR_melan- > Eumelanin_melan
Validation	Addition of EDN1 induced increase in tyrosinase activity in cultured human melanocytes [59].
**Biological Process Perturbed gene**	**UV induced melanin synthesis**
	**Tyr_melan**
Control Path	UV- > Singlet_oxygen_kerat- > Ceramide_kerat- > PLA2_kerat- > Arachidonic_Acid_kerat- > PGE2_kerat- > PTGER3_melan- > PLC_melan- > DAG_melan- > PRKCB_melan- > TYR_melan- > Melanogensis
Alternate Path	None
Validation	Tyrosinase mutations lead to albinism or hypopigmentation (MGI)
**Biological Process Perturbed gene**	**UV induced dendrite formation**
	**KIT_melan**
Control Path	UV- > Singlet_oxygen_kerat- > Ceramide_kerat- > PRKCZ_kerat- > NFKB1_kerat- > KITLG_kerat- > KIT_melan- > PIK3CA_melan- > RAC1_melan- > RAC1:PARD6A:CDC42_melan- > PRKCZ_melan- > Dendrite_formation_melan
Alternate Path	UVB- > Lipid_Peroxidation_kerat- > 4HNE_kerat- > DNA_Damage_kerat- > TP53_kerat- > ACTH_kerat- > MC1R_melan- > ADCY4_melan- > cAMP_melan- > RAP1A_melan- > RAC1_melan- > RAC1:PARD6A:CDC42_melan- > PRKCZ_melan- > Dendrite_formation_melan
Validation	Highly dendritic melanocytes are stimulated by injection of alpha-MSH in newborn mice. Formation and translocation of melanosomes to dendrites is triggered by alpha-MSH [60].
**Biological Process Perturbed gene**	**UV induced melanosme phagocytosis**
	**F2RL1_kerat**
Control Path	UV- > Singlet_oxygen_kerat- > Ceramide_kerat- > PRKCZ_kerat- > NFKB1_kerat- > F2RL1_kerat- > RHOA_kerat- > Melanosome_phagocytosis_kerat
Alternate Path	None
Validation	RWJ-50353, a serine protease inhibitor, led to reduced pigment deposition in melanocytes and de-pigmentation. Immature melanosomes accumulate inside melanocytes and there is abnormal dendrite dynamics in RWJ-50353-treated epidermal equivalents [61].
**Biological Process Perturbed gene**	**UV induced melanocyte proliferation**
	**NFKB1_kerat**
Control Path	UV- > Singlet_oxygen_kerat - > Ceramide_kerat- > PRKCZ_kerat- > NFKB1_kerat- > CSF2_kerat- > CSF2RA_melan- > CSF2RA:JAK1_melan- > STAT3_melan- > CCND1_melan- > CDK4:CCND1_melan- > Cell_proliferation_melan
Alternate Path	UVB- > Lipid_Peroxidation_kerat- > 4HNE_kerat- > DNA_Damage_kerat- > TP53_kerat- > ACTH_kerat- > MC1R_melan- > ADCY4_melan- > cAMP_melan- > PRKACA_melan- > CREB1_melan- > MITF_melan- > CDK4_melan- > CDK4:CCND1_melan- > Cell_proliferation_melan
Validation	In T-oligos pre-treated and UV light-irradiated keratinocytes, NFκB binding to the transcriptional co-activator p300 decreased relative to control whereas the amount of p53 binding to p300 was strikingly increased demonstrating activation of p53 and repression of NFkB upon DNA damage in keratinocytes which could serve as an alternate path when NFkB is perturbed [62].

Validation refers to observations from literature that could possibly support the existence of such pathways.

### New insights from the model

Data from the network shows the influence of signalling from multiple receptors in processes leading to pigmentation. Significant contributors to skin pigmentation have been identified by the model, with several receptors seen to be playing a key role. Receptors EGFR, F2RL1, FAS, IL1R1, IL6R, PTGER2, PTGER4 and TNFRSF1A in keratinocytes, and CSF2RA, EDNRB, FZD3, KIT, MET, NTRK1, PTGER1 and PTGER3 in melanocytes are observed to be important for pigmentation. MC1R signalling is seen to be important in both keratinocytes and melanocytes. Some of the paths in the model corroborate with knockout and siRNA data that emphasize the need for certain proteins for the manifestation of the skin pigmentation phenotype and their role in various skin pathologies. The model also highlights paracrine signalling in cells of the epidermis wherein factors secreted from keratinocytes in response to UV have a key role to play in adult skin pigmentation by regulating signalling in melanocytes. The model to a large extent encompasses signalling involving various physiological factors that are known to play a role in adult skin pigmentation such as MC1R, CREB, MITF, PAX3, SOX10, LEF1/TCF, F2RL1, SCF, HGF and GM-CSF [15].

## Discussion

Human skin responds to UV radiation exposure by increasing the expression of melanin in skin, a pigment that protects it from UV induced damage [6]. Melanin has a role to play in various pigmentation disorders such as albinism [20], vitiligo [21] as well as in melanoma [22] and non-melanoma skin cancers [23]. A detailed understanding of the process of skin pigmentation is thus vital for understanding the molecular pathology of these disease conditions. Melanin synthesis takes place in response to UV induced cues from neighbouring cells such keratinocytes and dermal fibroblasts [24], and exerts its protective effects when transported and expressed in keratinocytes [25]. Multiple roles are suggested for the protective effect of melanin in UV induced damage [26]. Melanin can filter UV radiation and scavenge reactive oxygen species produced as a result of UV exposure. The supra-nuclear cap of melanin protects cells from DNA damage. It is reported to inhibit production of cyclobutane pyrimidine dimers and 6,4-photoproducts, both of which are mutagenic [27]. Patients with albinism have been shown to be more susceptible to various skin cancers [28].

Given the fact that manifestation of the phenotype involves a complex cross-talk across different cell types that are present in different strata of the skin through a well regulated sequence of events, it becomes imperative to study the process from a systems perspective. The availability of data through several reductionist experimental studies over the years has greatly accelerated advances in systems’ analyses of pigmentation [29]. A recent study integrated functional genomics and protein-protein interaction networks to identify novel components that impact melanogenesis, focusing largely on the endothelin receptor (EDNRB) mediated signalling pathway [30]. Our study also supports the importance of ENDRB mediated signalling in melanocytes in the process of melanogenesis.

Our model describes a well annotated, curated and directed interaction network among 265 components that could have a direct or indirect role in influencing skin pigmentation. This is the first study reporting a directed interaction network of human skin pigmentation, highlighting the functional nature of interactions among different components, as well as the direction in which a signal has to flow to be biologically meaningful. Of the 265 nodes, 183 have a role to play in regulating more than one biological process influencing skin pigmentation. This reflects the complexity as well as the connectivity within the system where a single node can influence multiple biological processes. The model suggests that multiple cues from neighbouring cells can trigger similar processes in melanocytes, highlighting the various possibilities of multiple alternate paths a system could take leading to a common end process. The importance of certain nodes has been illuminated, some of which are well known, such as the indispensability of tyrosinase in melanogenesis [31] and the role of MITF in melanosome formation [32]. We also report a list of nodes that are essential for certain processes, the absence of which leads to abrogation of signalling through the required pathway. The model helps in broadening the perspective of known data by suggesting an extended signalling pathway or potential alternate paths in the absence of a node. F2RL1 is known to play an important role in melanosome transfer [33]. The model suggests that NFKB could be the transcription factor regulating the expression of F2RL1 in keratinocytes and hence NFKB could also have a key role to play in melanosome transfer. RAC1 is involved in MSH mediated dendrite formation on melanocytes [34]. The current model predicts the possibility of an alternate path involving paracrine signalling of KITLG from keratinocytes acting on the c-kit receptor on melanocytes leading to RAC1 activation and subsequent dendrite formation. The model reiterates the communication between keratinocytes and melanocytes.

These observations highlight the need to study biological processes in the context of the system as a whole instead of individual entities, as varied factors both external and internal have a role to play in their regulation. Perturbation studies with individual source-destination pairs enable the identification of components that are essential for a biological process or its phenotypic manifestation. It also hints at the possible dispensable nature of certain proteins and redundancy in the network by suggesting alternate signalling routes.

The vastness and constant addition of data in this field impose a practical limitation on model completeness. Though the network is extensive, it can be further extended with the availability of more information. The model can also be expanded to include factors from dermal fibroblasts, which are now being reported as increasingly important in the manifestation of pigmentation [35].

Aberrant melanin expression can lead to multiple pathological conditions like albinism - which results in a complete loss of pigmentation, vitiligo - wherein there occur hypo-pigmented patches of skin, sunburn and skin damage due to insufficient protection of the skin from UV radiation, or melanoma, one of the most aggressive forms of human skin cancer. Skin pigmentation has also been a significant field of interest for the cosmetic industry which attempts to address issues of lentigines/age spots and improving the skin tone. Use of anti-oxidants for correcting pigmentation disorders [36,37] can be readily elucidated from the model as UV induced oxidative stress is one of the primary steps triggering signalling leading to regulation of skin pigmentation. Thus, a foundation of cellular signalling has been provided which can be used to study conditions of disease and provide a basis for identifying therapeutic targets.

## Conclusions

Through this study we have presented a systems perspective to the process of UV mediated human skin pigmentation, identifying underlying pathways in the process. A comprehensive, well annotated directed interaction network of various molecules participating in the manifestation of skin pigmentation in the epidermis has been constructed. The model has provided a detailed description of signalling pathways triggered in epidermal cells, and sequential signalling in keratinocytes and melanocytes. Several plausible routes of biological signalling in the network have been identified, and the importance of certain components and their effects on signalling pathways has been ascertained. A comprehensive understanding of the molecular events leading to skin pigmentation can thus provide valuable inputs for identifying potential targets for therapeutic consideration as well as a rational basis for design/improvement of commercial products. The approach adopted can be applied to various biological systems and serves as a powerful tool to analyse cross-talk among various biological components.

## Methods

### Building a network model

Through an exhaustive literature survey, different processes in skin pigmentation including melanin biosynthesis, melanosome formation in melanocytes followed by transfer of melanin carrying melanosomes to keratinocytes via dendrites and phagocytosis in keratinocytes were identified. For each of these processes, important participating molecules including proteins, secondary messengers and small molecules were identified. A model describing these processes was built as a static map using the software Cell Designer (version 4.4) [38]. Based on this data, an interaction network was constructed for these molecules that participate in different stages leading to pigmentation. In the network, individual proteins and in some cases metabolites form the nodes and interactions among the nodes form the edges. The network included protein-protein, protein-small molecule and protein-biological process interactions. All protein-protein interactions were high confidence interactions extracted manually, or from the STRING database and NetPro™ [39]. The protein-small molecule interactions and protein-biological process interactions were curated from literature [approximately 100 articles from PubMed]. The interactions included in the network pertained primarily to human data but data from mouse/rat species was also used to fill in certain missing links. The network included molecular interactions influencing pigmentation in response to UV radiation in two primary epidermal cell types- keratinocytes and melanocytes. Interactions in the network were assigned direction, based on information available in literature and KEGG pathways. Directions were dependent on the functional nature of the interaction, for e.g. ‘phosphorylation’ or ‘activation’. All nodes were named according to their compartment, with the suffix kerat or melan ascribed to them (for e.g. NFKB_kerat), depending on their presence in keratinocytes or melanocytes. This compartment-specific interaction information was manually curated through literature.

### Network analysis

The constructed network was visualized in Cytoscape v.3.1.0 [40]. Network properties of node and edge centrality, degree distributions, radiality, and shortest path lengths were computed using the plugin NetworkAnalyzer. For computing shortest paths, Dijkstra’s algorithm implemented in Matlab using the Matlab Boost Graph Library [41] was used. Perturbation analysis for selected nodes was carried out by eliminating the chosen nodes and their corresponding edges specific to each perturbation.

## Additional files

Additional file 1:
**Directed interaction network of 265 nodes and 429 edges.** The functional 563 nature of interaction is also described. Sources from literature describing each interaction have been provided. Additional file 2:
**Network properties of the interaction network.** Computed network properties of degree, centrality and betweenness have been provided. Additional file 3:
**127 nodes in the S2T** **sub-network** **annotated according to the biological processes they participate in or influence.**
Additional file 4:
**Paths from source to target nodes in the S2T** **sub-network.** 157 computed shortest paths from 20 source nodes to 9 destination nodes have been enlisted. Additional file 5:
**Paths computed post perturbation analysis and identification of essential nodes.** Effects of perturbation of 33 nodes on source to target paths with possible alternate routes are provided. Essential nodes for each path have been enlisted.
